# RETRACTION: “AGEs/RAGE Promote Osteogenic Differentiation in Rat Bone Marrow‐Derived Endothelial Progenitor Cells via MAPK Signaling”

**DOI:** 10.1155/jdr/9897272

**Published:** 2026-11-04

**Authors:** Journal of Diabetes Research

**RETRACTION:** Y. Wang, C. Jiang, Z. Shang, G. Qiu, G. Yuan, K. Xu, Q. Hou, Y. He, and Y. Liu, “AGEs/RAGE Promote Osteogenic Differentiation in Rat Bone Marrow‐Derived Endothelial Progenitor Cells via MAPK Signaling,” *Journal of Diabetes Research* 2022, no. 1 (2022): 4067812, https://doi.org/10.1155/2022/4067812.

The above article, published online on 01 February 2022 in Wiley Online Library (http://wileyonlinelibrary.com), has been retracted by agreement between the journal Editor, Saba Noor and John Wiley & Sons Ltd. The retraction has been agreed after an investigation into figure concerns that were raised on PubPeer by *Xenogliocladiopsis eucalyptorum* and *Illex illecebrosus* [1]. Specifically, the GAPDH loading controls across Figure 7 (a) share unexpected similarities. In addition, a potential splice was detected in Figure 7 (a), blot “SB203580 – p‐p38MAPK”.

The authors had previously contacted the journal to request a correction to the GAPDH bands, which were assembled incorrectly during figure preparation. However, the authors were unable to provide all of their original, unedited, exposure files for the “SB203580” blots. As such, the editor no longer has confidence in the results and conclusions presented in this article.

The authors disagree with this decision.
